# Host gene effects on gut microbiota in type 1 diabetes

**DOI:** 10.1042/BST20220004

**Published:** 2022-05-06

**Authors:** Keyu Guo, Juan Huang, Zhiguang Zhou

**Affiliations:** 1 National Clinical Research Center for Metabolic Diseases, Key Laboratory of Diabetes Immunology (Central South University), Ministry of Education, and Department of Metabolism and Endocrinology, The Second Xiangya Hospital of Central South University, Changsha, China; 2 Section of Endocrinology, Department of Internal Medicine, School of Medicine, Yale University, New Haven, CT, U.S.A.

**Keywords:** gut microbiota, host gene, type 1 diabetes

## Abstract

Type 1 diabetes (T1D) is an organ-specific autoimmune disease characterized by progressive pancreatic β-cell loss. Both a predisposing genetic background, that may encompass mutations in several genes, as well as exposure to environmental factors can affect the progression of autoimmune responses to multiple pancreatic islet autoantigens. Many genetic variants that increase the risk of T1D are found in immunity genes involved in sensing and responding to microorganisms. Although increasing evidence indicates that the gut microbiome composition may promote or prevent T1D development, little is known about the link between gut microbiota and T1D susceptibility genes in patients with T1D. Recent studies in the inbred non-obese diabetic (NOD) mouse, a widely used model of T1D, have suggested that many genetic loci can influence gut microbiome composition to modulate islet autoimmunity. This review summarizes evidence that examines the effect of host genes on gut microbiota diversity and function during T1D development. Knowledge of the host gene-gut microbiota interactions at play during T1D progression may help us identify new diagnostic and prognostic tools and help also design effective strategies for disease treatment.

## Introduction

**Figure 1.**
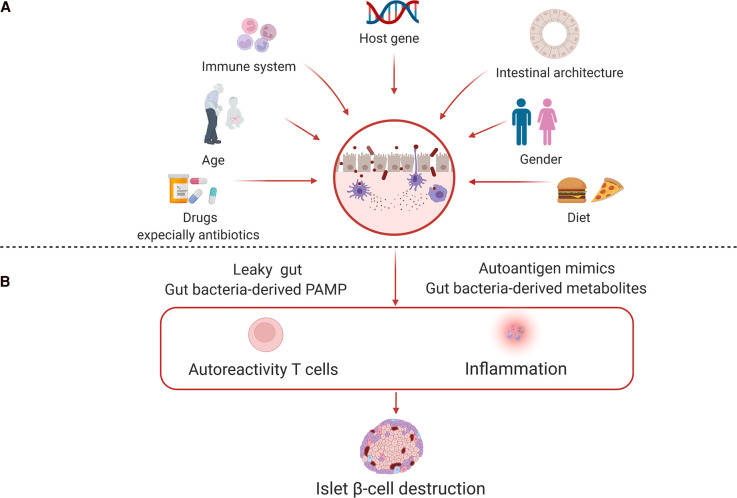
Major factors affecting gut microbiome composition and their roles in T1D development. (**A**) The composition of the gut microbiota is under the influence of a complex interplay between both host and environmental factors. (**B**) Dysbiosis of the gut microbiota has been implicated in islet autoimmunity through autoantigen mimicry, alterations in intestinal permeability, and generation of metabolites and pathogen-associated molecular patterns (PAMPs) that bind PRRs to activate autoreactive T cells. The ensuing inflammation contributes to the destruction of β-cells in the pancreatic islets.

Type 1 diabetes (T1D) is an autoimmune disease caused by the immune cell-mediated destruction of β-cells in the pancreatic islets [1]. Both genetic predisposition and environmental factors are involved in the development of T1D by compromising immune homeostasis [2]. Polymorphisms of the human leukocyte antigen (HLA) region within the major histocompatibility complex (MHC) have been recognized as major contributors to genetic susceptibility to the development of T1D [3]. In addition to HLA genes, multiple non-HLA loci affect immune regulation and peripheral immune tolerance, also increasing T1D risk [4]. Studies have also suggested that non-HLA risk genes are active not just in immune cells but also in islet β-cells, where they influence responses to innate stimuli, inflammation, and other stressors and may ultimately favor dysfunction and apoptosis of islet β-cells. Although genetic factors affecting T1D onset are well known, environmental factors that influence T1D development are still poorly defined. Several environmental exposures have been linked to T1D, including toxins, viral infections, dietary factors, and more recently the microbiome. Recently, the gut microbiota has emerged as a pivotal contributor to T1D progression [5]. Research has shown that individuals in early stages of T1D (those with islet autoimmunity) as well as after onset, display distinct gut microbial diversity, taxonomy abundance, and function compared with healthy control subjects [6–12]. Moreover, numerous studies have demonstrated that the gut microbiota plays an essential role in the modulation of host immunity and suggested its involvement in T1D progression [13–17] (Figure 1). As T1D is a multifactorial disease driven by both genetic predisposition and environmental factors, understanding the interaction between the host's genetics and its associated gut microbiota during T1D development should expand our knowledge about T1D pathogenesis.

Relationships between specific host genetic loci and gut microbiome composition have been unraveled by genetic association studies, microbiome genome-wide association studies (mGWAS), and investigations using engineered animal models [18]. Early insights into the interaction between the host genome and gut microbes were obtained from animal-based studies. These demonstrated that host genes related to immunity, metabolism, and behavior traits were associated with the relative abundance of specific microbial taxa [19]. In recent years, combined whole-genome DNA sequencing and mGWAS analyses identified genetic factors that affect the composition and functional diversity of the gut microbiome [20]. The first mGWAS study was conducted in 2015 and found 83 associations between host genetic polymorphisms and the abundance of specific bacterial taxa [21]. This study revealed that single nucleotide polymorphisms (SNPs) in the lactase gene LCT, associated with the lactase persistence phenotype, have the most powerful explanatory power to predict abundance of *Bifidobacterium* [21]. After that, several mGWAS studies further identified a number of genetic variants involved in the regulation of immunity, metabolism, and gut architecture, in association with a distinct composition of the gut microbiome [22–25]. Still, an obvious influence of genetic factors on gut microbiota composition is supported by human studies that revealed that monozygotic twins tend to have a more similar gut microbiome profile than dizygotic twins [26]. However, gut microbiome is affected by genetics, diet, lifestyle and other factors. Even monozygotic twins living in the same family environment may have very different gut microbiome suggesting that there are many unknown factors that play an important role in the shaping of gut microbiome. It is noteworthy that whereas in a natural primate population 97% of gut microbiome phenotypes were significantly heritable, in humans <20% of gut microbes were shown to have non-zero heritability [27]. This result is qualitatively different from all similar human studies so far, excluding the lack of environmental factors that may affect the level of heritability in cross-sectional studies, and shows that the almost universal heritability of gut microbiome, rather than only a few heritable phenotypes found in humans, emphasizing the importance of deep longitudinal sampling.

**Figure 2.**
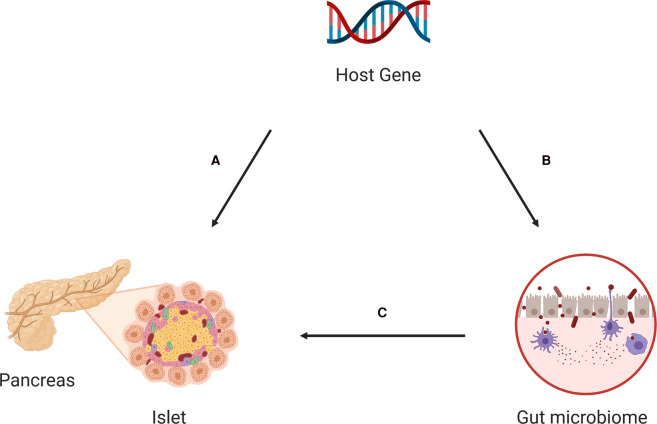
Potential host genetics-gut microbiome-islet autoimmunity interactions. (**A**) Host genetic polymorphisms may influence islet autoimmunity independently of host–microbiome interactions. (**B-C**) Host genetic polymorphisms may modulate microbiome composition (**B**), indirectly influencing islet autoimmunity (**C**).

Adding to the extensive research that unmasked potential links between human genetic variation and T1D susceptibility, recent studies in the inbred non-obese diabetic (NOD) mouse model of T1D suggested that many genetic loci can influence the gut microbiota composition (Table 1) [28–33]. This review summarizes relevant animal and human studies with the aim to elucidate potentially significant host gene effects on gut microbiota in T1D. We propose different scenarios by which host gene–microbiome interactions might modulate gut microbial dynamics and function to influence islet autoimmunity and disease development (Figure 2).

**Table 1:** Host gene effects on gut microbiota in NOD mice

Gene product	Experimental model	Main findings	References
MYD88	*Myd88*^−/−^ NOD mice	*Myd88* deficiency changed the composition of the distal gut microbiota, reduced the Firmicutes/Bacteroidetes ratio, and protected mice from T1D development. Transfer of gut microbiota from *Myd88*^−/−^ NOD mice reduced insulitis and delayed the onset of diabetes in WT NOD mice.	[28]
TRIF	*Trif*^−/−^ NOD mice	*Trif*^−/−^ NOD mice had a different microbiota composition compared with WT NOD mice, resulting in a reduced proinflammatory phenotype and function of immune cells associated with diabetes susceptibility.	[33]
NOD2	*Nod2*^−/−^ NOD mice	*Nod2*^−/−^ NOD mice had a distinct gut microbiome profile compared with *Nod2*^+/+^ NOD mice, which influenced immune cell function to modulate T1D susceptibility.	[31]
MHC molecules	*Eα16* transgene NOD mice	*Eα16* transgene NOD mice harbored a distinct microbiota profile. Transfer of microbiota from *Eα16* transgene NOD mice alleviated disease progression in WT NOD mice.	[32]
MHC molecules	Congenic NOD.H-2^b^ mice	Introduction of protective MHC alleles in NOD.H-2^b^ mice was associated relatively small overall changes to the microbiota along with decreased intestinal inflammation.	[30]
IL-2	Congenic NOD. *Idd3*/*Idd5* mice	Pathways linked to IL-2 signaling and immune regulation were related to microbial profile alterations in both NOD mice and humans. Diabetes-protected strains exhibited a restoration of immune regulatory pathways in the intestine, an effect mimicked by IL-2 therapy.	[30]
IL-10	*Il-10*^−/−^ BDC2.5^+^ NOD mice	*Il-10* deficiency in BDC2.5^+^ NOD mice increased susceptibility to islet autoimmunity through modulating neutrophil hemostasis, an effect accompanied by altered intestinal immunity and gut microbiome profile.	[29]

## MHC polymorphisms

Many of the T1D risk variants identified by GWAS are located in immunity genes involved in sensing and responding to microorganisms [25]. Alterations in MHC genes, represented by the HLA gene complex in humans, are considered the major genetic risk factors for T1D. These genes encode cell-surface glycoproteins that present antigens to T cells, and are thus essential to host immunity against extracellular microbes [34,35]. Available evidence shows that the primary mechanism by which MHC II regulates host–microbiota interactions is by regulating T cell-dependent anti-commensal IgA responses [34,36–41]. In turn, recent findings have suggested that MHC alleles are also associated with the diversification of the gut microbiome [42,43]. For instance, functional microbiome diversification among MHC congenic mouse strains was detected upon comparison of bacterial cellular fatty acid (CFA) profiles [44]. Additionally, Bolnick et al. [45] demonstrated that there is an association between certain MHC motifs and relative abundance of some microbial families in the stickleback fish (a classic model organism for studying MHC polymorphisms). Subsequently, an accumulating body of research on transgenic and congenic mice indicated that MHC genetics contributes to shaping the gut microbiome, influencing the genesis and development of diseases [32,44,46]. Meanwhile, a recent study quantifying the influence of HLA alleles on human gut microbiota reported that individuals with similar HLA genes have similar microbiota profiles [42]. A correlation between variants located in the HLA region and gut microbiome composition was also found in a mGWAS study [47]. Furthermore, a population-based study of 3002 subjects showed that individuals with functionally similar HLA haplotypes are more likely to harbor similar microbiome profiles [42]. As for autoimmune T1D, the All Babies in Southeast Sweden (ABIS) prospective cohort study demonstrated that HLA genetic risk for developing autoimmune T1D is associated with microbiota variance in the gut [48]. Moreover, immune responses to gut bacteria in individuals at T1D risk have been shown to be associated with pancreas islet autoimmunity and development of T1D in an HLA genotype-dependent manner [49]. Along these lines, it was reported that NOD mice expressing an Eα transgene (a diabetes-resistant MHCII allele) harbored a distinct microbiota profile [32]. Notably, transfer of microbiota from Eα transgene NOD mice into wild type (WT) NOD littermates remarkably alleviated disease progression in the recipients [32]. These results provide a reasonable basis for therapeutic protection against islet autoimmunity afforded by specific MHC alleles operating via the gut microbiome.

## Pattern-recognition receptor signaling-related alleles

Innate immunity plays an important role in defending against infection by sensing specific ligands derived from exogenous microorganisms to induce innate immune responses and shape adaptive immunity, eventually modulates β-cell autoimmunity[50,51]. Pattern recognition receptors (PRRs), an integral part of the innate immune system, act as a host protector from bacterial invasion via recognizing and binding bacterial components [52]. PRRs consist of Toll-like receptors (TLRs), nucleotide binding oligomerization domain-like receptors (NOD-like receptors, NLRs), scavenger receptors (SRs), C-type lectin receptors (CLRs), and β2-integrins [53]. Nucleotide-binding oligomerization domain-containing protein 2 (NOD2), an important member of the NLR family, functions as a cytosolic sensor of muramyl dipeptide, a component of bacterial cell walls [54]. A study of 474 individuals established a connection between relative abundance of *Enterobacteriaceae* and *NOD2* variants [55]. Studies using streptozotocin-induced diabetic C57BL/6 mice as well as *Nod2*^−/−^ NOD mice demonstrated that NOD2 plays a role in T1D development [31,56]. Consistent with previous research, compared with *Nod2*^+/+^ NOD mice, *Nod2*^−/−^ NOD mice showed decreased levels of antimicrobial defensins and displayed changed gut microbiota composition [31]. Furthermore, an increase in both secreted IgA and IgA-producing B-cells in Peyer's patches was detected in *Nod2*^−/−^ NOD mice [31]. In line with this observation, a significant increase in IgA transport was recently observed in patients with a *NOD2*-mutation [57]. The latter study, conducted in Crohn's disease patients, provides further evidence that NOD2 plays an important role in IgA transport through human and mouse M cells [57]. Importantly, *Nod2*^−/−^ NOD mice possess a different gut microbiome profile compared with *Nod2*^+/+^ NOD mice, showing also a modified immune cell function that impacts T1D susceptibility [31].

TLRs play critical roles in inflammatory pathways in response to microbial components and act also as modifiers of the susceptibility to autoimmune diseases [58,59]. In T1D studies, *Tlr2*, *Tlr7*, or *Tlr9* deficiency in NOD mice confers a protective effect on T1D development, whereas *Tlr4* deficiency correlates positively with the progression of the disease [60–62]. An mGWAS study demonstrated that the *TLR1* gene is related to the abundance of *Selenomonas* in the throat and of Lautropia in the tongue dorsum, which strengthens the potential relationship between *TLR* gene polymorphisms and gut microbiome composition [21]. Indeed, in T1D patients an association was found between *TLR2/TLR4* expression and relative abundance of *Bacteroidetes* and *Firmicutes* in the gut microbiota [63]. Myeloid differentiation primary response gene 88 (MyD88) and TIR-domain-containing adapter-inducing interferon-β (TRIF) are two major adaptor molecules in the TLR signaling pathway. Both *Myd88*^−/−^ and *Trif*^−/−^ NOD mice have a different microbiota composition compared with WT NOD mice, resulting in a reduced inflammatory immune response that is associated with reduced diabetes susceptibility [28,33]. However, transferring gut microbiota from WT NOD mice was shown to block the protective effect mediated by *Trif* or *Myd88* deficiency. Based on findings to date, we can conclude that allelic variation in PRRs signaling pathways modify the composition of the gut microbiota, which affects autoimmune diabetes occurrence and development by altering host immunity.

## Immunoregulatory cytokine loci

The cytokines IL-10 and IL-2 are key orchestrators of immune responses in the intestine [64,65]. In addition, recent GWASs also found association between *IL-10* and *IL-2* gene and T1D [66,67]. Our recent studies showed that *Il-10* deficiency in BDC2.5^+^ NOD mice alters both intestinal immunity and gut microbiome composition and increases susceptibility to islet autoimmunity through modulating neutrophil hemostasis [29]. We further showed that the effect of IL-10 on the homeostasis of neutrophils was indeed dependent on gut microbiota alterations [29]. However, findings regarding the role of IL-10 in T1D development are inconsistent [68,69]. In turn, it was reported that T1D-associated variants in IL-2 pathway genes can potentially lead to gut microbiome changes, promoting intestinal inflammation [48,70]. NOD mice carrying protective alleles at T1D susceptibility loci Idd3 (*Il-2*) and Idd5 (*Ctla4*, *Slc11a1*, and *Acadl*) have an altered microbiota profile compared with WT NOD mice. More importantly, a study in the TwinsUK cohort of human subjects confirmed that T1D-protective alleles linked to IL-2 pathway loci cause certain microbiota alterations similar to those observed in NOD mice [48].

## FUT2 genotype and secretor status

**Figure 3.**
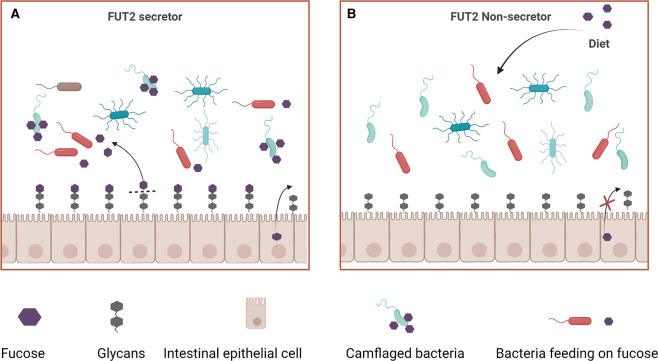
Role of the FUT2 genotype in shaping the gut microbiome. (**A**) In FUT2 secretors, fucose is a major bacterial energy source and allows specific gut microbes to evade host immunity. (**B**) In FUT2 non-secretors, no terminal fucose is added to mucosal glycans. FUT2 non-secretors have an altered, less diverse gut microbiome profile, which may be reestablished by dietary supplementation with polysaccharides.

The FUT2 gene codes for the enzyme α-(1, 2)-fucosyltransferase, which catalyzes the transfer of fucose to galactose terminal residues on N-glycans [71]. Fucose is an essential energy source for bacteria and raw material for synthesis of the bacterial capsule. Of note, fucose is used by Bacteroides species to colonize the gut and escape from the immune defense of the host [72,73]. Several polymorphisms have been reported in the *FUT2* gene. Individuals homozygous for the null *FUT2* allele lack terminal fucose residues on mucin glycans and are referred to as ‘non-secretors’, whereas individuals carrying at least one functional allele are defined as ‘secretors’, in reference to the absence and presence, respectively, of soluble ABO blood group antigens in bodily fluids such as saliva, urine, and tears [74]. Interestingly, gut microbial profiles vary considerably between *FUT2* non-secretors and secretors, with *FUT2* non-secretors having lower bacterial diversity and a significant decrease in the abundance of both *Bifidobacterium* and *Bacteroides* [75,76] (Figure 3). In *Fut2*-deficient NOD mice, it was suggested that gene effects on the composition of the gut microbiome can be mitigated by complex polysaccharide supplementation in diet [77]. *FUT2* gene has been linked to human T1D risk in GWAS study with genome-wide significance [67]. Although an association between FUT2 non-secretor status and T1D was reported in Caucasian and Japanese population samples [78,79], and children carrying the high-risk HLA genotype that are homozygous *FUT2* non-secretors showed predisposition to rapid disease progression [80], the actual role of *FUT2* gene variants in T1D is still disputed [71]. Therefore, further research is needed to clearly establish the effects of *FUT2* gene variants on gut microbiota composition and T1D development and progression.

Genetic factors provide susceptibility to diseases, while environmental factors are the triggers of T1D. Epigenetics is considered a bridge between genetic and environmental factors. Epigenetics can regulate the expression of key genes involved in the autoimmunity and β-cell vulnerability, which eventually lead to the autoimmune-mediated β-cell destruction and T1D [81]. At present, many studies focus on the regulation of gut microbiome on epigenetics. This review focuses on the role of host genetic factors in gut microbiome, further research still needs to explore the influence of epigenetics on gut microbiome.

## Conclusions

Growing evidence supports the notion that host genetics contributes to the composition of its associated microbiome, which carries important implications on host health and disease susceptibility. It has become clear that the HLA system in humans and the MHC II loci in animals represent one of the most important genetic factors influencing the host's microbiome. The corresponding alleles, which encode antigen presentation molecules, were shown to shape the host's ability to interact with the microbiota by regulating the recognition of gut microbes, altering the binding affinity toward bacterial antigens, and affecting the host antibody immune response.

Numerous studies, carried in animal models and T1D patients, validated the association between MHC/HLA genes and risk of T1D in relation to compositional changes in the gut microbiota. In turn, genetic variations impacting on PRR signaling pathways, immunoregulatory cytokines, and FUT2 expression and function have also been proposed to influence T1D susceptibility and/or contribute to disease severity and progression by altering gut microbiota diversity.

Further investigations addressing host gene–microbiome associations in T1D should provide valuable insights into T1D pathogenesis by clarifying the impact of host genetics and gut microbiota composition and function in relation to T1D risk and progression. Importantly, combining host and microbial genotyping, as well as metabolite profiling, might also drive the development of microbiome-based therapeutic strategies for T1D prevention and treatment.

## Perspectives

- Understanding the interactions between the microbiome and host genetics may provide valuable insights into T1D diagnosis, treatment, and prevention.
- Studies in NOD mice and T1D patients suggest that genetic variation in multiple loci (e.g. the MHC/HLA region, PRR receptors, immunoregulatory cytokines, and the FUT2 gene) influences gut microbiota composition, affecting T1D susceptibility and severity.
- Further studies are needed to uncover causal relationships between host genetics, especially MHC gene, and the gut microbiome and their role in the occurrence and development of T1D.

## Abbreviations

CFA: cellular fatty acid
CLRs: C-type lectin receptors
HLA: human leukocyte antigen
mGWAS: microbiome genome-wide association study
MHC: major histocompatibility complex
MyD88: myeloid differentiation primary response gene 88
NLRs: nucleotide binding oligomerization domain-like receptors
NOD: non-obese diabetic
PRRs: pattern recognition receptors
SNPs: single nucleotide polymorphisms
SRs: scavenger receptors
T1D: type 1 diabetes
TLRs: Toll-like receptors
TRIF: TIR-domain-containing adapter-inducing interferon-β
WT: wild type
